# Deep-Sea Hydrothermal Vent Viruses Compensate for Microbial Metabolism in Virus-Host Interactions

**DOI:** 10.1128/mBio.00893-17

**Published:** 2017-07-11

**Authors:** Tianliang He, Hongyun Li, Xiaobo Zhang

**Affiliations:** College of Life Sciences and Laboratory for Marine Biology and Biotechnology of Qingdao National Laboratory for Marine Science and Technology, Zhejiang University, Hangzhou, People’s Republic of China; CEH-Oxford

**Keywords:** deep-sea hydrothermal vent, marine virus, metabolic compensation, microbial metabolism

## Abstract

Viruses are believed to be responsible for the mortality of host organisms. However, some recent investigations reveal that viruses may be essential for host survival. To date, it remains unclear whether viruses are beneficial or harmful to their hosts. To reveal the roles of viruses in the virus-host interactions, viromes and microbiomes of sediment samples from three deep-sea hydrothermal vents were explored in this study. To exclude the influence of exogenous DNAs on viromes, the virus particles were purified with nuclease (DNase I and RNase A) treatments and cesium chloride density gradient centrifugation. The metagenomic analysis of viromes without exogenous DNA contamination and microbiomes of vent samples indicated that viruses had compensation effects on the metabolisms of their host microorganisms. Viral genes not only participated in most of the microbial metabolic pathways but also formed branched pathways in microbial metabolisms, including pyrimidine metabolism; alanine, aspartate, and glutamate metabolism; nitrogen metabolism and assimilation pathways of the two-component system; selenocompound metabolism; aminoacyl-tRNA biosynthesis; and amino sugar and nucleotide sugar metabolism. As is well known, deep-sea hydrothermal vent ecosystems exist in relatively isolated environments which are barely influenced by other ecosystems. The metabolic compensation of hosts mediated by viruses might represent a very important aspect of virus-host interactions.

## IMPORTANCE

Viruses are the most abundant biological entities in the oceans and have very important roles in regulating microbial community structure and biogeochemical cycles. The relationship between virus and host microbes is broadly thought to be that of predator and prey. Viruses can lyse host cells to control microbial population sizes and affect community structures of hosts by killing specific microbes. However, viruses also influence their hosts through manipulation of bacterial metabolism. We found that viral genes not only participated in most microbial metabolic pathways but also formed branched pathways in microbial metabolisms. The metabolic compensation of hosts mediated by viruses may help hosts to adapt to extreme environments and may be essential for host survival.

## INTRODUCTION

In deep-sea hydrothermal vents, the wide chemical and thermal gradients around the vent chimneys provide habitats for microorganisms and benthonic animals (1). After more than 40 years of studies on deep-sea hydrothermal vents, one of the most extreme ecosystems on earth, our understanding of microbial community structures in these extreme environments has been greatly expanded (2, 3). In the deep-sea hydrothermal vent ecosystem, chemolithoautotrophic microorganisms use chemical energy to synthesize organic matter and provide the primary nutrients for the whole ecosystem, thus having regulatory effects on deep-sea hydrothermal systems by serving as the basis of the food chain (4, 5). However, recent studies found that virus-like particles are more abundant than prokaryotes in deep-sea hydrothermal vent ecosystems and may have very important roles in regulating microbial community structure, the microbial food web, and the biogeochemical cycle (6–10).

Viruses are ubiquitous, most abundant, and highly genetically diverse in marine ecosystems (11, 12). Most marine viruses are bacteriophages, which are the major factors causing mortality of heterotrophic and autotrophic microorganisms (13). It has been demonstrated that phages can regulate abundance of their bacterial hosts (10), lyse host cells to control microbial population sizes (14), and affect community structures of hosts by killing specific microbes (15). In deep-sea hydrothermal vents, the mechanisms of lysis of thermophiles by bacteriophages have been explored (8, 16–18). The “kill-the-winner” model predicts that phages kill the dominant microbial strains to leave living space and nutrients for the strains which are resistant to them (10, 19). This model may explain the enormous diversity observed in microbial communities (20). Phages also affect microbial diversity by horizontal gene transfer (10). During assembly in donor host cells, phages package some part of host genes, which are then transferred and inserted into the genomes of recipient host cells in the next infection (21, 22). The differences of phage-packaged genes dramatically change the genotype and phenotype of the hosts (20). In the long-term interactions between phages and hosts, the host microorganisms can change their genomes to obtain phage resistance, while phages can also change their genomes to recover the ability to infect hosts for reproduction (23, 24). The antagonism between the phages and hosts increases the diversity of viral and microbial community structures (25).

It has been well recognized that the relationship between phages and host microbes is that of predator and prey (14). However, some recent studies reveal that phages may be essential to microbial survival (26, 27). Without a complete cellular structure, phages must utilize the replication and synthesis machinery of their host microbes to reproduce themselves (22). Because of the presence of these intracellular interactions, phages may provide significant benefits to their hosts (28, 29). The benefits can allow their hosts to absorb more nutrition and contribute to host survival in unfavorable environments (9). So far, it remains unclear whether the benefits (such as nutrition absorption and host survival) derived from phages by their hosts are an individual phenomenon or widely exist in phage-host interactions. As is well known, the deep-sea hydrothermal vent ecosystems exist in relatively isolated environments which are barely influenced by other ecosystems (30). Our previous study showed that thermophilic phages interacted with their host thermophiles in the deep-sea vent (8). In this context, this unique deep-sea vent ecosystem offers an excellent opportunity to explore whether the existence of phages is beneficial for deep-sea microbial survival and adaptation to environments.

To explore whether viruses are beneficial or harmful to their hosts, viromes without exogenous DNA contamination and microbiomes of three deep-sea hydrothermal vents at different geographical distances were analyzed in this study. The analysis revealed that viruses had compensation effects on microbial metabolisms in the deep-sea vent ecosystem.

## RESULTS

### Bacterial communities in deep-sea hydrothermal vents.

**FIG 1:**
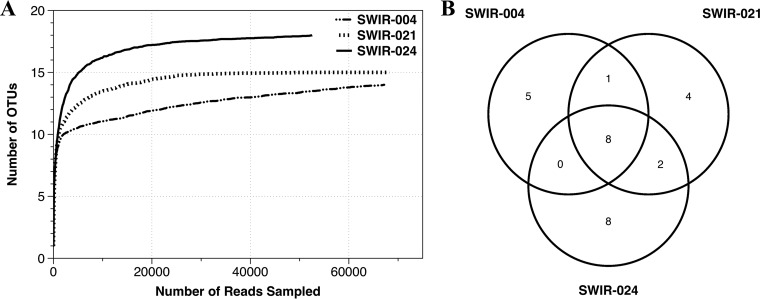
Bacterial OTUs (operational taxonomic units) for sediments from three deep-sea hydrothermal vents in the Southwest Indian Ocean. (A) Rarefaction curves of the bacterial 16S rRNA genes from three samples. (B) Venn diagram of bacterial OTU distributions in the three samples.

To explore the virus-microorganism interaction in deep-sea hydrothermal vents, the bacterial community structures of vents from the Southwest Indian Ocean were investigated. The pyrosequencing analyses for 16S rRNA genes of bacteria from three sediment samples (SWIR-S004, SWIR-S021, and SWIR-S024) yielded 197,640 reads and identified 28 operational taxonomic units (OTUs) (Table 1). Based on OTUs at 3% dissimilarity, the rarefaction curves were approaching plateau (Fig. 1A), and the library coverages of the three samples almost reached the maximum values (Table 1). To ensure the reliability of bacterial 16S rRNA gene sequencing, the DNA was reextracted from the sediment sample (SWIR-S004, SWIR-S021, or SWIR-S024) using the cetyltrimethylammonium bromide (CTAB) method and then the bacterial 16S rRNA gene sequencing was conducted for the second time. The pyrosequencing analyses indicated that the raw data of the second sequencing of bacterial 16S rRNA genes (NCBI accession no. PRJNA384468) were covered by those of the first sequencing (NCBI accession no. PRJNA309222), showing that the results of bacterial 16S rRNA gene sequencing were reliable and that there was no DNA contamination for the DNA extraction. These results indicated that most of the samples well represented the bacterial communities in the deep-sea vents.

**TABLE 1:** Analysis of bacterial 16S rRNA genes of deep-sea hydrothermal vent sediments

Sample	No. of sequences	97% similarity			
		No. of OTUs	Coverage (%)	Shannon (lci, hci)^a^	Simpson (lci, hci)^a^
SWIR-S004	67,173	14	99.69	1.21 (1.2, 1.22)	0.3891 (0.3861, 0.3921)
SWIR-S021	68,201	15	99.93	1.05 (1.05, 1.06)	0.4267 (0.4237, 0.4297)
SWIR-S024	52,546	18	99.96	1.16 (1.16, 1.17)	0.3901 (0.3877, 0.3926)

^a^ lci and hci indicate the lower and higher 95% confidence intervals, respectively.

**FIG 2:**
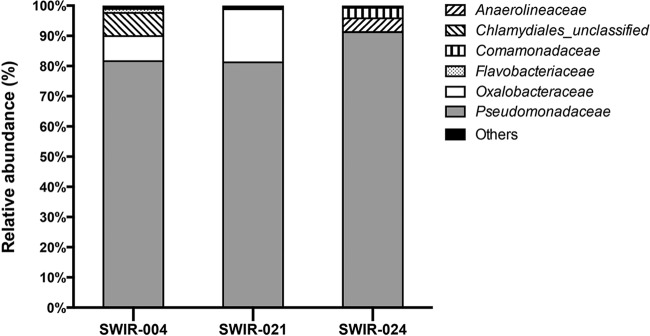
Relative abundance of bacterial families of sediments from three deep-sea hydrothermal vents in the Southwest Indian Ocean. The category “Others” represents the bacterial families with less than 1% of reads.

The 28 OTUs could be assigned into 9 phyla, 20 families, and 25 genera. *Pseudomonadaceae* were the most abundant OTUs, accounting for 81.49%, 80.97%, and 91.04% of reads in samples SWIR-S004, SWIR-S021, and SWIR-S024, respectively (Fig. 2). Comparison of the OTU distributions of the three samples showed that only 8 OTUs were shared by all three samples but represented 94.15% of all reads (Fig. 1B). Also, the Shannon and Simpson diversity indices showed less difference among the three samples (Table 1). These results indicated that similar bacterial community structures existed in samples SWIR-S004, SWIR-S021, and SWIR-S024.

### Viral and microbial metagenomes in deep-sea hydrothermal vents.

**FIG 3:**
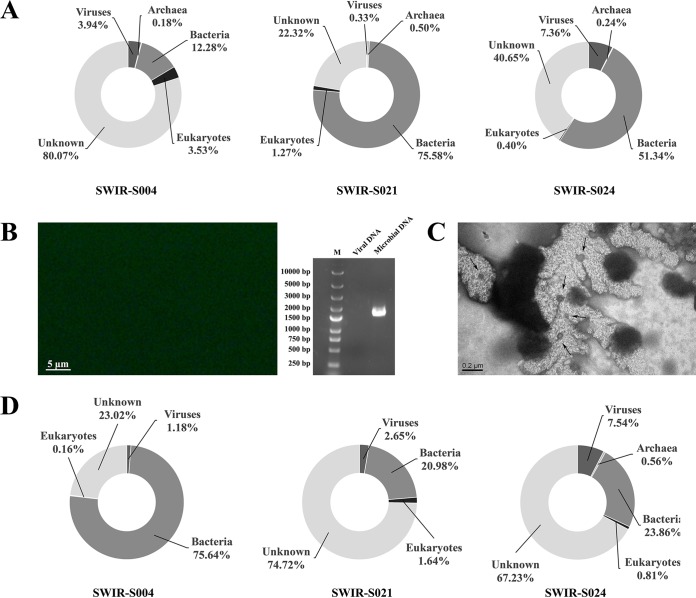
Viral and microbial metagenomes in deep-sea hydrothermal vents. (A) Taxonomic composition of the sequence contigs in SWIR-S004, SWIR-S021, and SWIR-S024 samples from deep-sea hydrothermal vents. (B) Examination of exogenous DNA contamination in the virome samples. The SYBR green-stained virome samples were examined by fluorescence microscopy (left). Bar, 5 μm. At the same time, the amplified bacterial 16S rRNA genes from the virome samples and the microbiome samples were analyzed by agarose gel electrophoresis (right). M, DNA marker. (C) TEM image of virus particles from deep-sea hydrothermal vent sediments. The arrows indicate the virus particles. Bar, 200 nm. (D) Taxonomic composition of sequence contigs in virome libraries (SWIR-S004, SWIR-S021, and SWIR-S024). The relative abundance of the sequence contigs was classified by the taxonomic grouping based on BLASTx similarity search (E value, <10^−3^).

To investigate the relationship between viruses and their hosts in deep-sea hydrothermal vents, the viromes and microbiomes from three sediment samples collected in the Southwest Indian Ocean (SWIR-S004, SWIR-S021, and SWIR-S024) were analyzed. To obtain sufficient sequencing quantity, 3 Gbp raw data for each microbial metagenome library was used. The raw data were assembled into contigs. Microbiome libraries of the three samples contained 9,129, 6,183, and 10,543 sequence contigs, respectively (Table 2). These sequence contigs were subjected to a BLAST search against the sequences deposited in the nonredundant protein (NR) database. The results indicated that most of the sequence contigs were significantly similar (E value, <10^−3^ in BLASTx) to the sequences of bacteria in the NR database, accounting for 12.28%, 75.58%, and 51.34% of all the sequence contigs in the SWIR-S004, SWIR-S021, and SWIR-S024 samples, respectively (Fig. 3A).

**TABLE 2:** Analysis of viral and microbial genomic data

Parameter	SWIR-S004		SWIR-S021		SWIR-S024	
	Virome	Microbiome	Virome	Microbiome	Virome	Microbiome
No. of reads	56,545,254	54,693,245	40,528,971	73,054,402	50,269,953	56,322,645
No. of contigs	2,634	9,129	3,241	6,183	14,624	10,543
GC content (%)	53.82	66.91	41.76	47.23	52.61	50.06
No. of matched contigs^a^	2,027	7,091	819	1,232	4,792	6,257

^a^ Matched contigs were evaluated based on BLASTx similarity search (E value, <10^−3^) to the sequences deposited in the nonredundant protein database.

In order to exclude the exogenous DNA contamination in the viromes, the virus particles in deep-sea vent samples were purified with nuclease (DNase I and RNase A) treatments and cesium chloride density gradient centrifugation. The results of epifluorescence microscopy of SYBR-stained virome samples showed that no fluorescent particle was observed in the virome samples (Fig. 3B). The PCR analysis of 16S RNA showed that 16S RNA was amplified in the microbiome samples but not in the virome samples (Fig. 3B). These results indicated that there was no exogenous DNA contamination in the virome samples. At the same time, the results of bacterial 16S rRNA gene sequencing revealed that there was no DNA contamination for the DNA extraction. The transmission electron microscopy (TEM) data showed that virions, possibly belonging to different virus groups, were isolated, indicating that the virus isolation method used in our study could effectively obtain virus particles from deep-sea vent sediments (Fig. 3C). To enrich the metagenomic DNA of viruses, the purified virions were subjected to genome amplification. The results showed that the amplified metagenomic DNA of virus could be used for metagenomic sequencing (see Fig. S1 in the supplemental material). The results excluded the exogenous DNA contamination of viral metagenomic DNA (Fig. 3B and C). To get sufficient sequencing quantity, 5 Gbp raw data for each viral metagenome library was obtained. Subsequently, the raw data were assembled to generate contigs. Based on sequencing, 2,634, 3,241, and 14,624 sequence contigs were obtained from the viromes of SWIR-S004, SWIR-S021, and SWIR-S024 samples, respectively (Table 2). After BLAST searching of the sequence contigs against the sequences of the NR database, it was revealed that lower proportions of sequence contigs were significantly similar to the sequences of viruses in the NR database, accounting for 1.18%, 2.65%, and 7.54% in SWIR-S004, SWIR-S021, and SWIR-S024, respectively (Fig. 3D).

10.1128/mBio.00893-17.1 FIG S1  Amplified viral metagenomic DNA. M, DNA marker. Download FIG S1, TIF file, 0.2 MB. Copyright © 2017 He et al. 2017 He et al. This content is distributed under the terms of the Creative Commons Attribution 4.0 International license.

### Functional genes of viromes and microbiomes from deep-sea hydrothermal vents.

**FIG 4:**
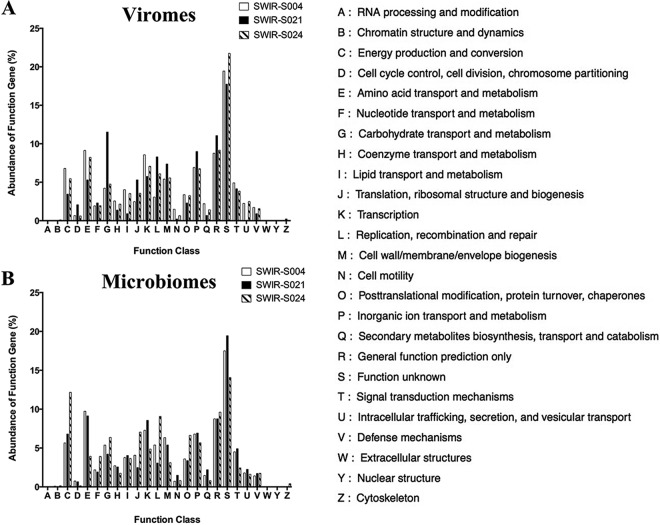
Functional genes of viromes and microbiomes from deep-sea hydrothermal vents. (A) Classification of predicted genes of viromes. (B) Classification of predicted genes of microbiomes. Each letter on the abscissa axis represents an eggNOG functional category. Genes with eggNOG orthology but without functional description were referred to as “function unknown.”

To characterize the functional genes of viromes and microbiomes from deep-sea hydrothermal vents, the sequences of viromes and microbiomes were analyzed by alignment against the eggNOG 4.5 database. The results indicated that 48.62% and 46.37% of the genes of viromes and microbiomes could be clustered into eggNOG categories, respectively. For all genes clustered into eggNOG categories, although the most abundant category was “function unknown,” many genes were classified in the categories of functional genes (Fig. 4A).

In the viromes, the genes involved in transcription, cell wall/membrane/envelope biogenesis, and inorganic ion transport and metabolism were included in the top five eggNOG categories for all three samples (Fig. 4A). Moreover, the genes related to “replication, recombination and repair” and “amino acid transport and metabolism” were more abundant, accounting for 3.07% to 8.29% and 5.3% to 9.13%, respectively, in SWIR-S004, SWIR-S021, and SWIR-S024 samples (Fig. 4A). These genes could function in the infection, DNA replication, assembly, and lysis of viruses. In all samples, the genes associated with cell metabolism, such as carbohydrate transport and metabolism; translation, ribosomal structure, and biogenesis; and secondary metabolite biosynthesis, transport, and catabolism, were abundant (Fig. 4A). These results reflected the fact that the viral genes of deep-sea vent viruses might participate in the cell metabolism of their hosts.

In the microbiomes, similar profiles of microbial functional genes were found in the SWIR-S004, SWIR-S021, and SWIR-S024 samples (Fig. 4B). The more abundant genes were those involved in energy production and conversion; amino acid transport and metabolism; transcription; replication, recombination, and repair; cell wall/membrane/envelope biogenesis; and inorganic ion transport and metabolism. These results indicated that the similar classification of functional genes of microbiomes existed in all three samples, despite the geographical distances between these samples (Table 3).

**TABLE 3:** Sampling information for deep-sea hydrothermal vents from Southwest Indian Ocean

Sample	Station position	Sampling date (mo/day/yr)	Depth (m)	Sample type
SWIR-S004	E 63.94°, S 27.85°	12/20/2013	2,958	Sediment
SWIR-S021	E 49.66°, S 37.88°	2/16/2014	2,219	Sediment
SWIR-S024	E 51.01°, S 37.55°	3/24/2014	2,400	Sediment

### Metabolic pathways of viromes and microbiomes.

**FIG 5:**
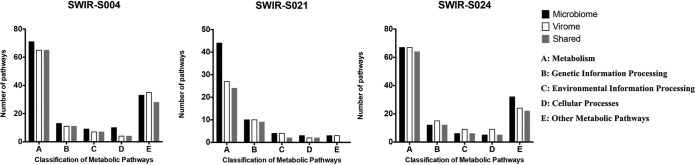
Classification of metabolic pathways of viromes and microbiomes from three samples. The classification of metabolic pathways was obtained according to KEGG pathway databases. “Shared” represents the metabolic pathways shared by the virome and microbiome.

All predicted genes from both viromes and microbiomes of deep-sea hydrothermal vents were aligned to KEGG pathways. The results indicated that the aligned metabolic pathways of microbial functional genes mainly were related to metabolism, genetic information processing, environmental information processing, and cellular processes in the SWIR-S004, SWIR-S021, and SWIR-S024 samples (Fig. 5). The three samples had similar classifications and abundances of metabolic pathways.

The metabolic pathways of viral functional genes which were involved in metabolism, genetic information processing, environmental information processing, and cellular processes were identical in the three samples (SWIR-S004, SWIR-S021, and SWIR-S024) (Fig. 5). The classification and abundance of pathways were the same among the three samples. The results showed that the classification of metabolic pathways in the virome was similar to that in the microbiome for the three samples, and most pathways were shared by the virome and microbiome (Table 4; Fig. 5). These shared pathways indicated that the virus genes might be involved in the microbial metabolic pathways.

**TABLE 4:** Analysis of KEGG pathways based on microbial and viral metagenomic sequences

Sample	No. of pathways		
	Microbial metabolic	Viral metabolic	Shared by viromes and microbiomes
SWIR-S004	146	122	115
SWIR-S021	64	46	37
SWIR-S024	122	124	109

### Metabolic compensation of viral genes in microbial metabolic pathways.

The analysis indicated that the viral genes participated in the metabolic pathways of microbes. It was found that there was metabolic compensation of viral unique genes in 6 microbial metabolic pathways including pyrimidine metabolism; alanine, aspartate, and glutamate metabolism; nitrogen metabolism and assimilation pathways of the two-component system; selenocompound metabolism; aminoacyl-tRNA biosynthesis; and amino sugar and nucleotide sugar metabolism.

**FIG 6:**
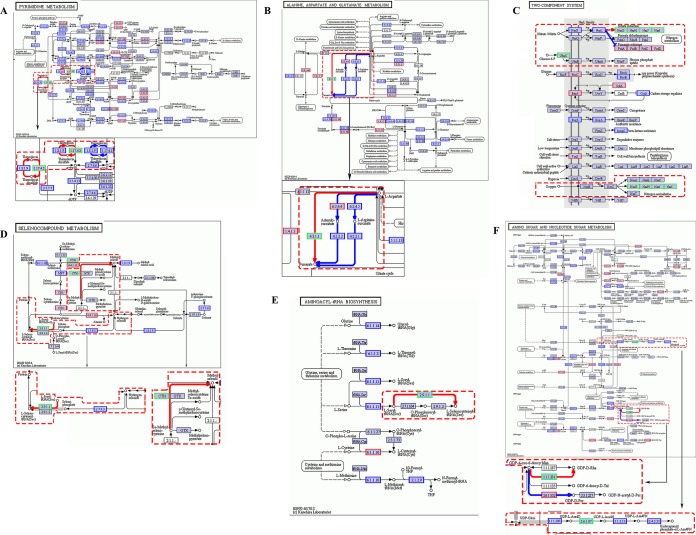
Metabolic compensation of viral genes in microbial metabolic pathways. (A) Pyrimidine metabolism pathway of microbes compensated by viral genes. EC 1.8.1.9, thioredoxin reductase; EC 1.17.4.1, ribonucleotide reductase, class II; EC 1.17.4.2, ribonucleoside-triphosphate reductase. (B) Microbial l-aspartate metabolism compensated by virus. EC 4.3.1.1, aspartate ammonia-lyase; EC 4.3.2.1, argininosuccinate lyase; EC 4.3.2.2, adenylosuccinate lyase; EC 6.3.4.4, adenylosuccinate synthase; EC 6.3.4.5, argininosuccinate synthase. (C) Metabolic compensation of viral genes in microbial two-component regulatory system. NarG, NarH, NarJ, and NarI, four subunits of nitrate reductase; NarX, nitrate-nitrite sensor histidine kinase; NarL, nitrate-nitrite response regulator; UhpC, sugar phosphate sensor protein. (D) Requirement of viral genes in selenocompound metabolism of microorganisms. CTH, cystathionine gamma-lyase; EC 2.9.1.1, l-seryl-tRNA (Ser) selenium transferase. (E) Crucial role of viral genes in aminoacyl-tRNA biosynthesis of microbes. EC 2.9.1.1, l-seryl-tRNA (Ser) selenium transferase. (F) Requirement for viral genes in amino sugar and nucleotide sugar metabolism. EC 2.6.1.102, perosamine synthetase; EC 1.1.1.281, GDP-4-dehydro-6-deoxy-d-mannose reductase; EC 2.6.1.87, UDP-4-amino-4-deoxy-l-arabinose-oxoglutarate aminotransferase. In all panels, the red, green, blue, and black boxes represent microbial genes, viral genes, genes shared by microbe and virus, and genes undetected in our work, respectively. The pathway compensated by virus is indicated with a dashed box.

As shown in Fig. 6A, ribonucleoside-triphosphate reductase (EC 1.17.4.2) derived from viruses was involved in pyrimidine metabolism of microbes. Thioredoxin and thioredoxin-disulfide were mutually transformed by thioredoxin reductase (EC 1.8.1.9) and ribonucleotide reductase, class II (EC 1.17.4.1), in microorganisms. The results showed that the viral ribonucleoside-triphosphate reductase (EC 1.17.4.2) could also transform thioredoxin to thioredoxin-disulfide, forming the branched pathway (Fig. 6A). This branched pathway mediated by viral genes could release more UTPs and CTPs for microbial pyrimidine metabolism, which might enhance the adaptability of microbes to deep-sea environments.

In microbial l-aspartate metabolism, l-aspartate could be catalyzed to generate fumarate through two pathways. One pathway was that l-aspartate was catalyzed by adenylosuccinate synthase (EC 6.3.4.4) to form adenylosuccinate, which was further catalyzed by adenylosuccinate lyase (EC 4.3.2.2) to generate fumarate (Fig. 6B). Another pathway involved the catalysis of l-aspartate by argininosuccinate synthase (EC 6.3.4.5) and subsequent catalysis of l-argininosuccinate by argininosuccinate lyase (EC 4.3.2.1). The results showed that the viral aspartate ammonia-lyase (EC 4.3.1.1) could directly transform l-aspartate to fumarate, which was more efficient for l-aspartate metabolism mediated by microbial enzymes (Fig. 6B). In l-aspartate metabolism of microbes, the virus compensated a novel metabolic pathway.

The metabolic compensation of viral genes in microbial metabolic pathways was also found in the microbial two-component regulatory system (Fig. 6C), which was a basic stimulus-response mechanism that allowed microorganisms to sense and respond to changes in many different environmental conditions. The two-component system of microbes is typically comprised of a membrane-bound sensor histidine kinase that senses extracellular signal molecules and cytoplasmic response regulatory proteins that regulate differential expression of target genes (31). The results revealed that the microbe sensor kinase NarX sensed extracellular nitrate or nitrite and then activated the response regulator NarL, leading to the gene expression inhibition of microbial fumarate reductase, including FrdA, FrdB, FrdC, and FrdD subunits in microbial nitrogen metabolism (Fig. 6C). However, the viral nitrate reductase consisting of NarG, NarH, NarJ, and NarI subunits could be activated by NarL to mediate microbial nitrogen metabolism (Fig. 6C). The viral nitrate reductase could also be activated by oxygen and then function in microbial nitrogen assimilation (Fig. 6C). It was revealed that the sugar phosphate sensor protein UhpC, which was derived from virus, was conducive to hexose phosphate uptake for microorganisms (Fig. 6C). These findings showed that viruses played very important roles in the microbial two-component regulatory system in terms of metabolic compensation.

The analysis showed that there was no microbial gene involved in transforming Se-methyl-l-selenocysteine to methylselenol and transforming l-seryl-tRNA (Sec) to l-selenocysteinyl-tRNA (Sec) in selenocompound metabolism of microbes (Fig. 6D). These two transformations could be completed by viral cystathionine gamma-lyase (CTH) and viral l-seryl-tRNA (Ser) selenium transferase (EC 2.9.1.1), respectively (Fig. 6D). These findings indicated that viruses were required to compensate microbial pathways in metabolism of microorganisms.

In aminoacyl-tRNA biosynthesis of microorganisms, l-seryl-tRNA (Sec) was catalyzed by viral l-seryl-tRNA (Ser) selenium transferase (EC 2.9.1.1) to generate l-selenocysteinyl-tRNA (Sec) (Fig. 6E). The data revealed that viral genes played crucial roles in microbial metabolism.

The compensation of viruses in microbial metabolisms was found in amino sugar and nucleotide sugar metabolism of microorganisms (Fig. 6F). In GDP sugar metabolism, the microbial perosamine synthetase (EC 2.6.1.102) could catalyze GDP-4-dehydro-6-deoxy-d-mannose (GDP-4-oxo-6-deoxy-Man) to generate GDP-4-amino-4,6-dideoxy-alpha-d-mannose (GDP-d-Per). However, the viral GDP-4-dehydro-6-deoxy-d-mannose reductase (EC 1.1.1.281) transformed GDP-4-dehydro-6-deoxy-d-mannose (GDP-4-oxo-6-deoxy-Man) to GDP-6-deoxy-d-mannose (GDP-d-Rha) (Fig. 6F). The viruses provided a branched pathway for GDP sugar metabolism in microbes. In the pathway through which UDP-glucuronate (UDP-GlcA) was transformed to 4-deoxy-4-formamido-alpha-l-arabinopyranosyl di-trans,octa-cis-undecaprenyl phosphate (undecaprenyl phosphate alpha-l-Ara4FN), microorganisms lacked the UDP-4-amino-4-deoxy-l-arabinose-oxoglutarate aminotransferase (EC 2.6.1.87) to catalyze UDP-beta-l-threo-pentapyranos-4-ulose (UDP-l-Ara4O), which must be completed by the viral UDP-4-amino-4-deoxy-l-arabinose-oxoglutarate aminotransferase (EC 2.6.1.87) (Fig. 6F), showing the requirement for viruses in microbial metabolism.

Taken together, these findings showed that the microbial metabolic compensation mediated by viral genes played key roles in the metabolism of microorganisms, which was formed during the long-term processes of virus-microorganism interactions in the deep-sea hydrothermal vents.

## DISCUSSION

To date, the virus-host interactions have been intensively explored. It is believed that the relationship between viruses and their hosts is generally a predator-prey relationship (14). During virus infection, viruses are known to regulate host macromolecular synthesis by modifying host transcription and translation machinery and making hosts serve the requirements of viruses (8, 32). Viruses are considered to be the crucial contributors to their hosts’ mortalities in the virus-host interactions. Viruses can shape their hosts’ community structure and function by destroying the hosts’ cells, while hosts can develop the immune systems to fight against viral invasion. In this study, however, the findings revealed that viruses had compensation effects on their hosts’ metabolism. This metabolic compensation of hosts mediated by viruses might be an important contributor to host survival in the long-term interactions between viruses and host organisms. Our study showed that viral genes participated in the hosts’ metabolism by forming branched pathways. These branched metabolic pathways mediated by viruses facilitated their hosts’ adaptation to various environments, thus being helpful to the hosts’ survival. In the present study, the viruses and hosts were from deep-sea hydrothermal vents. Given that the deep-sea hydrothermal vent ecosystems exist in relatively isolated environments which are barely influenced by other ecosystems (30), the virus-mediated metabolic compensation of hosts revealed in this study might represent an important aspect of virus-host interactions and might be ubiquitous in all ecosystems.

The microbial community structure in deep-sea vent ecosystems is influenced by many factors. Due to different environmental conditions, spatial differences of microbial diversity can exist in different deep-sea hydrothermal vents (5, 33, 34). In this study, the bacterial 16S rRNA gene sequencing of the SWIR-S004, SWIR-S021, and SWIR-S024 samples identified 28 OTUs. The number of OTUs in our study was lower than those in previous studies (35, 36). In our study, the rarefaction curves were approaching plateaus (Fig. 1A) and the library coverages of the three samples had reached the maximum values (Table 1), suggesting that the sequencing data were reliable. To further confirm the reliability of bacterial 16S rRNA gene sequencing, the DNA was reextracted from SWIR-S004, SWIR-S021, or SWIR-S024, followed by the second sequencing of the bacterial 16S rRNA genes. The results revealed that the bacterial 16S rRNA gene sequences of the second sequencing were included in those of the initial sequencing. In this context, the results of bacterial 16S rRNA gene sequencing were reliable in the present investigation. The differences in the number of OTUs and the dominant bacterial communities between this study and the previous study (37) might result from the different sampling times. The results of the present study showed that similar bacterial communities existed in the three samples (SWIR-S004, SWIR-S021, and SWIR-S024), despite the geographical distances between them. *Pseudomonadaceae* was the dominant bacterial family in all three samples.

In deep-sea hydrothermal vents, marine viruses regulate microbial diversity and abundance and impact global biogeochemical cycles by lysing their hosts (10). However, our data showed that viruses had compensation effects on microbial metabolism in the deep-sea vent ecosystems. High frequencies of prophages in deep-sea vents have been found (38, 39), suggesting that infection by viruses may facilitate their hosts’ survival. Some studies also reveal that viruses can function as key factors for their hosts’ survival in unfavorable environments (26, 27, 40). These results are consistent with our findings in this study. As reported previously, viruses can carry a variety of auxiliary metabolic genes to participate in their hosts’ metabolism (9, 41, 42). Lindell et al. (40) have showed that cyanophages carry the *psbA* gene encoding the photosystem II core reaction center protein D1. The essential photosynthesis protein encoded by cyanophages may be used to maintain energy generation after the host cell has ceased to manufacture photosynthesis proteins (43, 44). In *Escherichia coli*, prophages can provide a wider tolerance to environmental stressors despite not being inducible (45). It is reported that 15 double-stranded DNA viruses, which putatively infect sulfur-oxidizing bacteria, contain auxiliary metabolic genes for α and β subunits of reverse dissimilatory sulfite reductase (27). All the documented findings indicate that viruses can enable hosts to cope with different environmental conditions and that virus infection can provide significant benefits to virus survival and reproduction (26).

The results of this study present the compensation roles of viruses in microbial metabolic pathways. In future investigations, the dynamic influences of virus infection on microbial metabolism needed to be further explored. The heterogeneities of microbial functional genes and metabolisms at different stages of virus infection should be studied to reveal the virus-mediated metabolic compensation of hosts.

## MATERIALS AND METHODS

### Sample collection.

Samples were collected in the Southwest Indian Ocean during the Oceanic Vessel No. 1 cruises from December 2013 to March 2014 (Table 3). Sampling of sediments was conducted using sealable sampling boxes. To prevent mixing with seawater upon arrival on deck, the surface sediments of sediment samples were removed using sterile shovels. The samples were stored at −20°C until the microbial and viral DNAs were extracted.

### DNA extraction of microbes and viruses.

Sediment sample (20 g) was added to 200 ml of prefiltered (0.015-μm pore size) Milli-Q water and incubated for 30 min. After incubation, all samples were shaken for 10 min and then centrifuged at 500 × *g* for 1 min. The supernatant was filtered through a 0.22-μm tangential flow filter (TFF; Millipore, Westborough, MA, USA). The filtrate and the retentate were collected in separate tanks.

The microorganism-containing retentate was used for the extraction of microbial DNA. Microbial DNA was extracted from microorganism-containing retentate using the Power Water DNA isolation kit (Mo Bio Laboratories, USA). To exclude DNA contamination, the microbial DNA was also extracted from microorganism-containing retentate by the cetyltrimethylammonium bromide (CTAB) method (46). For each sample, DNA was extracted three times and then the DNAs were pooled. The resulting DNA was stored at −20°C until use.

The virus-containing filtrate was incubated with DNase I (1 mg/ml) and RNase A (1 mg/ml) for 1 h at room temperature and then supplemented with polyethylene glycol 6000 (PEG 6000) at a final concentration of 10% (wt/vol), followed by incubation for 18 h at 4°C. Virus particles were pelleted by centrifugation for 2 h at 50,000 × *g*. The virus pellet was resuspended in prefiltered Milli-Q water to a final volume of 2 ml. Subsequently the virus suspension was centrifuged at 220,000 × *g* with a cesium chloride density gradient (1.3 to 1.7 g/ml). The 1.5-g/ml fraction was collected. To exclude microbial contamination, the purified virions were stained with 2.5× SYBR Green I solution (BioTeke, Beijing, China) for 10 min in the dark, followed by examination under a Nikon Eclipse Ti-S epifluorescence microscope with a 100× high-resolution oil lens. At the same time, the bacterial 16S rRNA genes of the purified virions were amplified by PCR using the universal bacterial primer set 27F (5′-AGAGTTTGATCCTGGCTCAG-3′) and 1492R (5′-GGTTACCTTGTTACGACTT-3′). The PCR products were separated on a 1.2% agarose gel. The purified virions were examined under a JEM-1230 transmission electron microscope (TEM) operating at 120 kV. The viral DNA was isolated by formamide lysis and the CTAB method (46). For each sample, DNA was extracted three times. The pooled DNA was stored at −20°C for later use.

### Sequencing of bacterial 16S rRNA genes.

The microbial DNA extracted with the Power Water DNA isolation kit (Mo Bio Laboratories, USA) or the CTAB method was subjected to bacterial 16S rRNA gene sequencing. The microbial DNA was amplified using the universal bacterial primer set 338F (5′-ACTCCTACGGGAGGCAGCA-3′) and 806R (5′-GGACTACHVGGGTWTCTAAT-3′) covering the V3-V4 regions of the 16S rRNA genes. As a negative control, the sterile water was included in the microbial DNA amplification assay. The size of amplicons was 468 bp. PCR was carried out in a 20-μl reaction volume containing 4 μl 5× TransStart FastPfu buffer, 10 ng DNA template, 250 μM deoxynucleoside triphosphates (dNTPs), 0.2 μM (each) primer, and 2.5 U TransStart FastPfu polymerase (TransGen Biotech, China). PCR was conducted with initial denaturation at 95°C for 3 min and subsequently 27 cycles of denaturation at 95°C for 30 s, annealing at 55°C for 30 s, and extension at 72°C for 45 s, followed by a final extension at 72°C for 10 min. For an individual sample, three independent PCRs were performed to avoid bias. The PCR products for each sample were pooled. After separation on a 2% agarose gel, the PCR products were purified using the AxyPrep DNA gel extraction kit (Axygen, China) and quantified using the QuantiFluor-ST fluorescence quantitative system (Promega, CA, USA).

The bacterial 16S rRNA gene sequencing was performed by Shanghai Majorbio Bio-pharm Technology Co., Ltd. (China). The PCR products were used to prepare the barcoded library using the Illumina Nextera XT DNA sample preparation kit (Illumina, USA). Libraries were quantified on the Perkin Elmer LabChip GX (PerkinElmer, USA), normalized, and pooled at an equimolar ratio. Paired-end sequencing (2 by 300 bp) was performed using the MiSeq reagent kit v3 (Illumina, USA) according to the standard MiSeq protocol (Illumina, USA). PhiX spike was added at 5% concentration as recommended by Illumina (USA) for low-diversity libraries.

### Sequencing of microbial and viral metagenomes.

The viral DNA or microbial DNA was amplified using the GenomiPhi V2 DNA amplification kit, which can amplify whole-genome DNA, according to the manufacturer’s instructions (GE Healthcare Life Science, Buckinghamshire, UK). The template DNA was heated at 95°C for 3 min and then cooled at 4°C. Subsequently, the cooled DNA was mixed with reaction buffer and enzyme mix, followed by incubation at 30°C for 1.5 h to amplify the DNA. After amplification, the Phi29 DNA polymerase was inactivated at 65°C for 10 min and cooled at 4°C. For each sample, the viral DNA or microbial DNA was amplified three times and then the DNAs were pooled. The resulting metagenomic DNA was pyrosequenced using Illumina HiSeq 2000 by Shanghai Majorbio Bio-Pharm Technology Co., Ltd.

For the metagenomic DNA sequencing, the DNA was checked on a 1% agarose gel, purified using the AxyPrep DNA gel extraction kit (Axygen, China), and quantified with the QuantiFluor-ST fluorescence quantitative system (Promega, CA, USA). DNA shearing was conducted using an M220 focused ultrasonicator (Covaris Inc., Woburn, MA), and then 300-bp fragments were excised and extracted. The paired-end library was prepared with the TruSeq DNA sample prep kit (Illumina Inc., San Diego, CA) according to the standard protocol. Dual-index adapters containing the full complement of sequencing primer hybridization sites were ligated to the blunt-end fragments. Following amplification (10 cycles) and denaturation with sodium hydroxide, libraries were pooled and loaded onto an Illumina cBot. Paired-end sequencing (2 by 101 bp) was performed on an Illumina HiSeq 2000 system (Illumina Inc., San Diego, CA) at Majorbio Bio-Pharm Technology Co., Ltd. (Shanghai, China), using the TruSeq PE cluster kit v3-cBot-HS and the TruSeq SBS kit v3-HS according to the manufacturer’s manual.

### Data analysis.

For the bacterial 16S rRNA gene sequencing, the paired-end reads were overlapped to assemble the V3-V4 tag sequences using the Flash program (47). The percentage of sequences which could be paired was 80.52%. The primers and spacers were trimmed. The low-quality fragments and the sequences shorter than 50 bp were removed to minimize the effects of random sequencing error. The remaining sequences were further denoised and screened for chimeric sequences with the pre.cluster command and chimera.uchime command in Mothur (48). The candidate sequences were classified into operational taxonomic units (OTUs) by 97% sequence similarity using the Usearch program (49). The Shannon and Simpson diversity indices and rarefaction curves were generated using the Mothur program (48). Venn diagrams were implemented using the R package VennDiagram program.

For the microbial and viral metagenome sequencing, raw sequence reads of microbial and viral metagenomic sequencing were first trimmed to remove the reads of adapters and duplicate reads. The reads with a minimum length of 50 bp were subjected to *de novo* contig assembly using the SOAP assembly software (50) with the criterion of a 90% minimum overlap identity. The assembly data were aligned with sequences in the NCBI nonredundant nucleic (NT) database and the nonredundant protein (NR) database using BLASTn and BLASTx, respectively. The taxonomies of the aligned reads with the best BLAST value (E value, ≤10^−3^) were selected and used for further grouping analysis.

### Gene function and metabolism pathway annotation.

The putative amino acid sequences which were translated from assembled sequences were aligned against the eggNOG v4.5 (51) and KEGG (release 59.0) (52) databases using BLASTP (WU-BLAST 2.0; E value, ≤1e−5). Each predicted protein was assigned to the eggNOG orthologue group (OG) by the highest-scoring annotated hit(s) containing at least one high-scoring pair (HSP) scoring over 60 bits. KEGG annotation was performed with KOBAS 2.0 with default parameters in single best hit mode. The predicted proteins with KEGG orthology group (KO) were mapped at the KEGG Pathway.

### Accession number(s).

The nucleotide sequences of bacterial 16S rRNA and raw sequence reads of microbial and viral metagenomics reported in this study have been submitted to the Sequence Read Archive (SRA) of the NCBI database under the BioProject ID PRJNA309222. The nucleotide sequences of bacterial 16S rRNA for the second sequencing have been deposited in the SRA of the NCBI database under accession number PRJNA384468.
