# Supplementary figures and images for: Deep-Sea Hydrothermal Vent Viruses Compensate for Microbial Metabolism in Virus-Host Interactions
Source: mBio. 2017 Jul 11;8(4):e00893-17. doi: 10.1128/mBio.00893-17 (PMC5513705; doi:10.1128/mBio.00893-17)

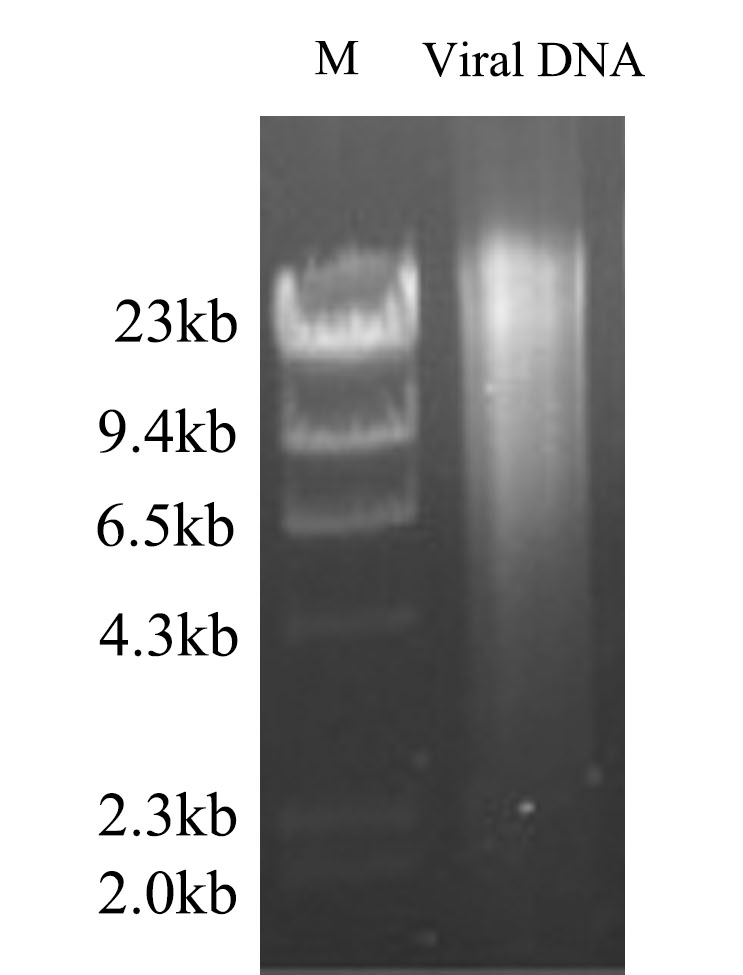

Supplement: FIG S1 [file mbo003173380sf1.tif]
